# Addressing low-value pharmacological prescribing in primary prevention of CVD through a structured evidence-based and theory-informed process for the design and testing of de-implementation strategies: the DE-imFAR study

**DOI:** 10.1186/s13012-020-0966-3

**Published:** 2020-01-22

**Authors:** Alvaro Sanchez, Jose Ignacio Pijoan, Susana Pablo, Marta Mediavilla, Rita Sainz de Rozas, Itxasne Lekue, Susana Gonzalez-Larragan, Gaspar Lantaron, Jon Argote, Arturo García-Álvarez, Pedro Maria Latorre, Christian D. Helfrich, Gonzalo Grandes

**Affiliations:** 1 Primary Care Research Unit, BioCruces Bizkaia Health Research Institute, Basque Healthcare Service, Osakidetza, Plaza Cruces s/n, E-48903 Barakaldo, Spain; 2 Clinical Epidemiology Unit, Hospital Universitario de Cruces. BioCruces Bizkaia Health Research Institute, Basque Healthcare Service-Osakidetza, Plaza Cruces s/n, E-48903 Barakaldo, Spain; 3 Primary Care Pharmacy Unit, Ezkerraldea-Enkarterri-Cruces Integrated Health Organization – Osakidetza, Plaza Cruces s/n, E-48903 Barakaldo, Spain; 4 Primary Care Pharmacy Unit, Barakaldo-Sestao Integrated Health Organization–Osakidetza, Antonio Miranda Etorbidea, E-48902 Barakaldo, Spain; 5 Department of Health Science Library, Cruces University Hospital-Osakidetza, Biocruces Bizkaia Health Research Institute, Plaza Cruces s/n, E-48903 Barakaldo, Spain; 6 Healthcare Integration Directorate, Ezkerraldea-Enkarterri-Cruces Integrated Health Organization–Osakidetza, Plaza Cruces s/n, E-48903 Barakaldo, Spain; 7 Healthcare Integration Directorate, Barakaldo-Sestao Integrated Health Organization–Osakidetza, Antonio Miranda Etorbidea, E-48902 Barakaldo, Spain; 8 Seattle-Denver Center of Innovation for Veteran-Centered and Value-Driven Care VA Puget Sound Health Care System, Seattle, USA; 9 Department of Health Services, University of Washington School of Public Health, Seattle, USA

**Keywords:** Low-value care, De-implementation, Cardiovascular disease prevention

## Abstract

**Background:**

De-implementation or abandonment of ineffective or low-value healthcare is becoming a priority research field globally due to the growing empirical evidence of the high prevalence of such care and its impact in terms of patient safety and social inefficiency. Little is known, however, about the factors, barriers, and facilitators involved or about interventions that are effective in promoting and accelerating the de-implementation of low-value healthcare. The De-imFAR study seeks to carry out a structured, evidence-based, and theory-informed process involving the main stakeholders (clinicians, managers, patients, and researchers) for the design, deployment, and assessment of de-implementation strategies for reducing low-value pharmacological prescribing.

**Methods:**

A phase I formative study using a systematic and comprehensive framework based on theory and evidence for the design of implementation strategies—specifically, the Behavior Change Wheel (BCW)—will be conducted to design and model de-implementation strategies to favor reductions in low-value pharmacological prescribing of statins in primary prevention of cardiovascular disease (CVD) by main stakeholders (clinicians, managers, patients, and researchers) in a collegiate way. Subsequently, a phase II comparative hybrid trial will be conducted to assess the feasibility and potential effectiveness of at least one active de-implementation strategy to reduce low-value pharmacological prescribing of statins in primary prevention of CVD compared to the usual procedures for dissemination of clinical practice guidelines (“what-not-to-do” recommendations). A mixed-methods evaluation will be used: quantitative for the results of the implementation at the professional level (e.g., adoption, reach and implementation or execution of the recommended clinical practice); and qualitative to determine the feasibility and perceived impact of the de-implementation strategies from the clinicians’ perspective, and patients’ experiences related to the clinical care received.

**Discussion:**

The DE-imFAR study aims to generate valid scientific knowledge about the design and development of de-implementation strategies using theory- and evidence-based methodologies suggested by implementation science. It will explore the effectiveness of these strategies and their acceptability among clinicians, policymakers, and patients. Its ultimate goal is to maximize the quality and efficiency of our health system by abandoning low-value pharmacological prescribing.

**Trial registration:**

Clinicaltrials.gov identifier: NCT04022850. Registered 17 July 2019

Contributions to the literature

- The DE-imFAR study is among the first studies applying the most prominent implementation theories and frameworks specific to the field of de-implementation.
- This study will use a systematic and comprehensive framework based on theory and evidence for the design and deployment of de-implementation strategies for reducing low-value statin prescribing in the cardiovascular disease primary prevention practice of clinicians
- This study will examine the feasibility potential effectiveness of the de-implementation strategy emerging from the aforementioned systematic and theory-based processes in reducing low-value statin prescribing as compared to usual procedures based on the principles of linear dissemination of “what-not-to-do” guidelines and recommendations

## Background

For a considerable proportion of interventions provided to patients in healthcare settings, there is a lack of solid evidence of their effectiveness (for example, pharmacological prescribing, programs and services). This implies that patients may receive ineffective, unnecessary or even harmful care. Additionally, a large part of healthcare spending is attributable to low-value care practices [1, 2]. The elimination or reduction of this low-value care can lead to improvements in the quality of care and its results in terms of population health while reducing costs. Nonetheless, while de-implementation of low-value care has received attention in the literature [3–5] and much is known about general barriers to reducing low-value care (e.g., cognitive bias [6], guidelines focused on underuse not overuse [7], and fragmentation of care [8, 9]), there is still great uncertainty about which interventions are effective in which contexts for promoting the abandonment of which types of low-value care [10].

For example, cardiovascular disease (CVD) remains one of the leading causes of premature death in Europe, accounting for 42% of deaths among women and 38% among men under 75 years of age. According to the World Health Organization, lifestyle changes would prevent more than three-quarters of cardiovascular deaths [11]. Statins are among the most prescribed medications globally for CVD prevention and are increasingly used in people without CVD or diabetes (“primary prevention”). The consumption of lipid-lowering drugs (statins) in the Basque Country (Spain) has gone from 46 defined daily doses/1000 inhabitants/day (DIDs) in 2006 to 90 DIDs in 2013, representing a growth of 93% [12]. This trend is despite statins having no added value for the primary prevention of CVD or at least having shown to be only effective or have some benefit for a small fraction of the target population depending on their baseline risk [13, 14]. Moreover, statins have associated side-effects (e.g., muscle pain) in 5 to 10% of users [15]. Meanwhile, healthy lifestyle promotion interventions in clinical settings have shown to be more cost-effective and are the preferred recommended practice, especially in low-risk patients. The prescribing of statins in low-risk populations (< 10%) can be considered a low-value practice [13, 14].

Evidence from studies of approaches to improving the adequacy and/or promoting the reduction or abandonment of low-value pharmacological prescribing shows, on the one hand, that training in isolation does not have an effect in terms of de-implementing inappropriate prescribing. On the other, multi-component interventions that combine multiple tailored interventions (audit and feedback, alert systems and clinical decision support systems) have achieved more positive results [16–23]. Nevertheless, there is no “magic bullet” to reduce clinicians’ use of low-value practices [24, 25]. From the growing scientific evidence in implementation research and the few theoretical advances and studies focused specifically on de-implementation, we propose that the following three intervention components are fundamental to successfully achieving the complex process of de-implementing a low-value practice.

First, it is known that involving the main stakeholders in a collaborative reflexive process allows a better understanding of the context and the implementation process, which in turn facilitates the identification of more successful implementation strategies that ensure feasibility. This procedure is commonly known as participatory action research [26]. Additionally, the application of targeted or adapted interventions maximizes the chance of success. Such tailored strategies are designed to improve healthcare based on an assessment of the determinants of the practice in question in a specific context [27, 28].

Second, we must bear in mind that de-implementation or unlearning is a process of abandoning or renouncing knowledge, values or behavior acquired, unconsciously or deliberately [29]. Unlearning can be difficult both individually and at the organizational level due to the possible “discomfort” that it generates [29, 30]. It may require losing faith in preexisting mental schemes in favor of new ones, opening the door to uncertainty, fear of the unknown and loss of a sense of control, with the consequent emotional impact that often leads to “defensive” postures [31]. It may also simply be difficult to suppress mental schemes that have guided a clinician’s decision-making and become second nature, even when the clinician is aware that these are no longer effective [32, 33]. The way in which such modes of thinking influence behavior and clinical decisions has implications for understanding how to effectively de-implement a given low-value practice. In relation to this, recent models based on unlearning and substitution suggest that clinical decision making is the result of two different modes of cognitive processing [30]: (1) intuitive cognition or type 1 system, which is a set of processes, largely unconscious, that occur in response to environmental or emotional cues and are based on previously learned ingrained heuristics; and (2) reflexive cognition or type 2 system, which is a conscious process of evaluating options based on a combination of utility, risk, capacities, and social influences. Unlearning is similarly difficult for organizations [29, 30], which are usually hierarchical systems characterized by a “dominant culture,” preconceived collective ideas, norms, and group expectations, that can obstruct both individual and organizational change. We propose that de-implementation efforts will therefore often require a multilevel approach. Further, it should be noted that de-implementation and implementation efforts are inherently linked as a reversal of disproven practices may require some sort of replacement with the desired effective practice [34].

Finally, improving the implementation of evidence-based practice by clinicians depends, in most cases, on enacting change in the behaviors involved. “Behavior change interventions” can be defined as a set of coordinated actions designed to change specific behavior patterns. But for these interventions to be effective, rather than be based on intuitive methods, they must be designed following a formal analysis process of the objective behavior and its theoretically foreseen mechanisms of action, all guided by models, theories or frameworks that cover the entire range of possible influences or determinants of behavior [35, 36].

Two of the most commonly used implementation frameworks are the Consolidated Framework for Implementation Research (CFIR) and the Theoretical Domains Framework (TDF) [37–39]. The CFIR [39], which focuses on determinants, includes 39 constructs structured into five domains (intervention characteristics; inner setting; outer setting; characteristics of the individuals; and the implementation process) regarding potential barriers to and facilitators of successful implementation. The TDF establishes a comprehensive systematic method for designing implementation strategies that were developed using a process of expert consensus and validation to identify psychological and organizational theories relevant to changing the clinical behavior of health professionals [37]. A set of 14 domains covering the main factors that influence clinical behavior were identified: knowledge; skills; role and social/professional identity; beliefs about capabilities; beliefs about the consequences; motivation and objectives; memory, attention and decision processes; environmental context and resources; social influences; emotion; behavior regulation; and the nature of the behaviors. These 14 domains provide a broad framework that covers most potential barriers to change, and therefore implies a long range of possible intervention components. To select the behavior change techniques most likely to produce change in a specific clinical behavior, the TDF and the associated Behavior Change Wheel (BCW) propose a step-by-step process to map the factors grouped by TDF domains, with an extensive [36] hierarchically organized taxonomy of 93 different behavioral change techniques, with definitions and clear examples that offer a reliable method to determine the active content of interventions.

Considering all this, the DE-imFAR (from the Spanish for DE-implementation of low-value pharmacological prescribing) study aims to carry out a structured, evidence-based, and theory-informed process involving the main stakeholders (managers, professionals, patients, and researchers) for the design and deployment of targeted de-implementation strategies for reducing low-value pharmacological prescribing, compared to the usual system-level procedures based on the principles of linear dissemination of “what-not-to-do” guidelines and recommendations. The DE-imFAR study will focus on the de-implementation of low-value pharmacological prescribing in primary prevention of CVD in patients at low cardiovascular risk, where the prescribing of statins is not indicated according to evidence-based clinical guidelines; and in the promotion of a healthy lifestyle as the core recommended intervention that will be considered the replacement component [26].

## Methods

### Aims

1. To design and model in a collaborative way among the stakeholders involved (healthcare professionals, patients, managers, and researchers) a set of de-implementation strategies to favor the reduction and/or abandonment of low-value prescribing of statins in primary prevention of CVD. This strategy will be designed using a systematic, comprehensive framework based on theory and evidence for the design of implementation strategies (the CFIR, TDF, and BCW) focused on addressing the main determinants (barriers and facilitators) of clinical practice for primary prevention of CVD and adapted to the specific context of primary care (PC) in the Basque Health Service (Osakidetza).
2. To evaluate the feasibility and potential effectiveness of the de-implementation strategies applied for reducing the low-value prescribing of statins in primary prevention of CVD and for increasing the delivery of healthy lifestyle promotion, in accordance with recommended clinical practice, compared to the usual procedures of dissemination of clinical practice guidelines, focused on the provision of educational materials, support tools, and training.

### Design

Implementation trial in phase I (formative mixed methods research) and phase II (feasibility and potential effectiveness evaluation using mixed methods). The DE-imFAR study was reviewed and approved by the Clinical Research Ethics Committee of the Basque Country (Ref: PI2019102, approved on 10 April 2019) and has been registered in the US NLM’s clinical trials database (clinicaltrials.gov NCT04022850, 17 July 2019).

Osakidetza provides universal coverage that is free at the point of use, aside from co-payment for drugs, funded through regional general taxation. Primary, specialized, and social health-related service provision is organized around 13 Integrated Healthcare Organizations (IHOs) that cover the three provinces of the Autonomous Community of the Basque Country: Araba, Bizkaia, and Gipuzkoa. Each resident is on the list of one family physician (FP) or pediatrician who offers comprehensive primary care and refers patients for hospital and specialty services. Primary care professionals work in full-time teams, including FPs, pediatricians, nurses, and administrative staff based at local centers providing access to healthcare for users in a defined geographical area.

#### Phase I formative mixed methods research

With the aim of designing and packaging context-specific de-implementation strategies to favor a reduction in low-value pharmacological prescribing in CVD primary prevention, the following actions will be carried out:

##### Cross-sectional observational study of inappropriate prescribing of statins in primary prevention of CVD

With the objective of quantifying and describing the magnitude and distribution of the problem regarding low-value pharmacological prescribing in CVD primary prevention, an observational descriptive study regarding inappropriate prescribing of statins in the last 5 years by Osakidetza PC physicians will be carried out. To this end, a clinical scenario in which the prescribing of statins is clearly inappropriate will be established. Specifically, the study population will consist of all patients attending PC consultations in the 2014–2018 period, aged between 40 and 74 years in men and between 45 and 74 years in women with hypercholesterolemia (LDL cholesterol > 70–189 mg/dL and/or total cholesterol > 200–289 mg/dL), but no known cardiovascular disease and an estimated cardiovascular risk < 5% according to the REGICOR scale [13]. The proportion of patients with and without an inappropriate prescription of statins as well as the annual incidence rate of new inappropriate prescriptions will be estimated both overall and by physician and IHO. Additionally, PC physicians will be classified into groups of low, moderate, and high rates of inappropriate prescribing. The data will be extracted from the electronic medical record system of Osakidetza (OSABIDE), at all times satisfying the legal and ethical requirements of anonymity and confidentiality.

##### Literature review: determinants of and effective intervention strategies to modify low-value pharmacological prescribing in CVD primary prevention

In order to describe the current state of knowledge in relation to factors that determine low-value pharmacological prescribing in the primary prevention of CVD, two areas will be reviewed: (a) determinants of pharmacological prescribing behavior in the context of CVD primary prevention, paying special attention to factors (personal, interpersonal, organizational, etc.) that favor/hinder low-value pharmacological prescribing; and (b) effective individual/organizational intervention strategies for the reduction, abandonment, or de-implementation of low-value pharmacological prescribing. To achieve this, a systematic search will be carried out in the MEDLINE, EMBASE, and Cochrane Library databases, for research published in the last 10 years. Terms will be used referring to the behavior of interest (pharmacological prescribing of statins or drugs for the reduction of cholesterol levels), to factors associated with prescribing at the level of professional, patient, or organization, to interventions to encourage abandonment or adaptation of the pharmacological prescribing according to practice recommendations (e.g., inappropriate prescribing), and to the type of study (e.g., descriptive or qualitative studies on determinants or associated factors; interventional studies or clinical trials to identify effective interventions). The following will be excluded: studies concerning the dissemination of clinical practice guidelines or recommendations without a systematic search process, reviews of such studies, and associated analysis of evidence; studies not performed in a routine clinical context with characteristics similar to that of Osakidetza; and studies conducted in the context of secondary or tertiary prevention.

The search will not be limited to any language or country. In addition to searching these databases, the references of all studies selected will be consulted to identify additional potentially eligible studies. The starting point of the search has been set as 2009 in order to focus on relatively recent studies that may better represent current working conditions, trends, and procedures in pharmacological prescribing. Two team members will independently review the abstracts and make an initial selection of studies identified by each search strategy. The final selection will be made by consensus after discussion of potential inconsistencies. The key information (methodological features, main results, quality of the research, and the evidence) for each of the studies selected in each of the aforementioned literature searches will be extracted using data forms and assessed.

##### Collaborative design and mapping of the de-implementation strategies

A working group will be created, composed of experts in the design of implementation strategies, methodologists, pharmacists, qualitative researchers, clinicians, and health service managers. The group will carry out a structured and theory-informed process to map implementation strategies seeking to address the determinants of low-value pharmacological prescribing in CVD primary prevention. More specifically, they will follow the process outlined by the BCW [35, 36] to identify, select, adapt, and define possible behavioral change interventions operationalized as de-implementation strategies to address the prioritized determinants of low-value pharmacological prescribing in CVD primary prevention. This process involves eight steps grouped into three stages:

*First stage—understand the behavior*: (1) define the problem in behavioral terms; (2) select the target behaviors; (3) specify the target behaviors; and (4) identify what has to change. To this end, two main actions will be carried out:

First, a semi-structured interview with a sample of FPs will be conducted to identify and break down the chain of behaviors and concomitant non-behavioral (e.g., contextual) factors that define the overall behavioral scenario. Three members of the working group, with different backgrounds, will independently review the recordings of the FP interviews and identify and propose a set of possible target behaviors and the complete behavioral scenario composed of the chain of micro-behaviors and concomitant factors. After that, they will agree on a single definition of the problem in behavioral terms. Subsequently, based on the information compiled from the interviews and using matrices and exercises proposed by the BCW, the working group will specify and select the final target behaviors most likely to lead to the desired behavior change, to be the focus of the next steps. Discrepancies will be resolved through discussion until an agreement is reached. The result of this analysis will be shown to the interviewees in order to validate the response and increase the level of consensus.

Second, a qualitative study using focus groups will be conducted to identify the determinants (barriers/enablers) of each of the target behaviors previously identified and selected. An intentional sample of FPs will be recruited, stratified by the pharmacological prescribing rate (low, moderate, and high) based on the descriptive study. To ensure that all perspectives are represented, sampling will continue until saturation, understood as two consecutive focus groups in which no additional material is gathered. The main goal of this qualitative inquiry process will be to identify the main barriers and facilitators for each of the selected target behaviors related to the provision of recommended clinical practice for primary prevention of CVD (e.g., intervention to promote healthy lifestyles for primary prevention of CVD) and those related to low-value pharmacological prescribing practice.

The groups will be led by two researchers with experience in qualitative research methods, as well as knowledge of the clinical field and the study objectives. The focus groups will be audio-recorded, with prior consent, and verbatim transcribed. The script of the focus groups will explore in detail potential determinants with questions formulated to cover each of the TDF and CFIR domains [37–39]. Both the TDF and CFIR are comprehensive multi-level implementation frameworks based on theory and evidence. The rationale for using both frameworks is their ability to identify a broad range of determinants, especially at the individual level with the TDF, and organizational level with the CFIR. This script will be developed by researchers with experience in behavioral change and implementation research, and clinicians with experience in the primary prevention of CVD in primary care settings. The script and operative procedures for the discussion groups will be piloted and refined, prior to the fieldwork. The data will be analyzed using an iterative process in which two researchers will perform an independent review and coding of the transcripts. Transcripts will be imported into the Atlas Ti program to manage the data and facilitate analysis. Any coding discrepancies will be discussed until consensus is reached.

Additionally, seeking to explore the determinants of low-value pharmacological prescribing practice in CVD primary prevention related to other stakeholders, these being patients in the target population, primary care nurses and cardiologists, qualitative inquiry processes will be performed based on discussion groups or key-informant interviews. As with physician groups, these qualitative procedures will be recorded and verbatim transcribed and analyzed. Informed consent of all participants will be obtained prior to these qualitative research procedures.

*Second stage—identify intervention options*: (5) select intervention functions; (6) select the specific behavior change techniques.

In order to identify the behavior change techniques most likely to produce changes for each of the identified determinants of selected target behaviors, the working group will proceed to map the barriers and facilitators identified and grouped in the TDF and/or CFIR domains, with behavior change strategies, intervention functions, and policy categories using the process established by the BCW [35, 36]. For all the range of possible interventions identified through this structured mapping process, the following will be set out: clear definitions of the techniques to be applied, the actual content of the interventions, their possible formats, and modes of execution, etc. A similar process for identifying potential implementation strategies will be also conducted using the CFIR-ERIC Implementation Strategy Matching Tool [40]. Convergences or divergences regarding strategies that emerge from the aforementioned processes will be resolved by consensus.

*Third stage—identify implementation procedures*: (7) select strategies and intervention techniques; (8) select the mode of execution of the intervention.The final selection of previously identified de-implementation strategies will be performed through a participatory consensus process involving the working group as representatives of the main stakeholders. In short, the working group will carry out a structured group process based on the nominal group technique to rank each of the tentative de-implementation strategies based on the APEASE criteria: affordability, practicability, effectiveness and cost-effectiveness, acceptability, side effects/safety, and equity. Strategies considered both highly feasible and relevant for enacting behavior change will be included the final set of specific strategies to be contained in at least one broad de-implementation strategy seeking to reduce low-value pharmacological prescribing in the primary prevention of CVD.

#### Phase II feasibility and potential effectiveness evaluation

A comparative hybrid type II feasibility/potential effectiveness implementation trial will be designed and conducted for evaluating at least one active de-implementation strategy defined through the phase I formative mixed research compared with the usual procedures of dissemination of clinical practice guidelines (e.g., “what-not-to-do” guidelines), focused on the provision of materials, support tools, and training (reference group). A mixed-methods evaluation will be undertaken: quantitative for assessing the results of implementation at the professional level (process and implementation outcomes regarding reductions in low-value pharmacological prescribing and the adoption, reach and implementation of the recommended clinical practice in CVD primary prevention) and qualitative for assessing the feasibility and perceived impact of the de-implementation strategy from the clinicians’ perspective and ascertaining the experience and satisfaction of patients concerning the clinical care received. The unit of intervention will be the PC physician, while observation and analysis will be performed at professional and patient levels. Thus, the trial will tentatively be randomized at the level of PC physician with at least two intervention arms (dissemination vs. de-implementation strategy). The diffusion of the final design of the planned evaluation is warranted.

### Participants

(i) Professionals: Osakidetza PC physicians willing to be involved in a process focused on the optimization of CVD primary prevention; and (ii) patients: all women aged 45–74 years and men aged 40–74 years with hypercholesterolemia but without diagnosed ischemic heart or cardiovascular disease and with a low estimated CVD Risk (< 5%) according to REGICOR who attend at least one appointment at the collaborating PC practices during the study period. Informed consent of PC physicians as representatives of the clusters will be obtained prior to trial commencement.

### Comparison de-implementation strategies

The active comparator will be passive dissemination, defined as a set of usual procedures for the dissemination of clinical practice guidelines (“what-not-to-do” guidelines) focused on the diffusion of knowledge and abilities mainly through the provision of materials, support tools, and training. This control intervention will be compared with at least one active experimental de-implementation strategy, emerging from the formative research, defined by a set of de-implementation strategies targeting the facilitators of the non-desired behavior (seeking to suppress it), while tackling the barriers to and encouraging the preferred/desired behavior. This experimental de-implementation intervention could tentatively be broken down into several distinct de-implementation strategies to be compared to the control intervention.

### Randomization

The FPs will be randomly assigned to one of at least two study arms, using a random number sequence generated by computer prior to the start of the trial: one of the groups will be exposed to the de-implementation strategy that emerges from the systematic process of identifying determinants and mapping tailored interventions established by the BCW; and the other the usual procedures for disseminating clinical practice guidelines (“what-not-to-do” recommendations) focused on the provision of materials, support tools, and training. The allocation sequence will be generated using a specifically restricted scheme by one member of the research team using PROC PLAN/alternatives. The sequence will be concealed at the coordination center. The physician will be allocated only after they have given written informed consent. The random assignment in a tentative three-arm design will be similar. Given that the strategy is an optimization of services or clinical care already offered in Osakidetza, participants are expected to be blind to the allocation group. The data analyst and the personnel in charge of measurements will be blind to physician allocation to study arms.

### Outcome measures

To assess the feasibility/potential effectiveness of compared de-implementation strategies in terms of public health significance, we will use the Reach, Efficacy, Adoption, Implementation, and Maintenance (RE-AIM) framework [41].

#### Reach

Percentage of eligible patients (low-risk patients with hypercholesterolemia but without ischemic heart or cardiovascular disease) ascribed to collaborating FP who received the recommended CVD primary prevention practice as dictated by the CVD Prevention Clinical Practice Guideline [13], this being the health promotion intervention action (e.g., assessment of advice regarding healthy lifestyles), 12 months after exposure of the FP to the compared de-implementation strategies

#### Effectiveness

Changes observed in attitudes towards health promotion and perceived ability of physicians to provide a health promotion intervention considered the recommended practice for the primary prevention of CVD in low-risk patients, measured through the Preventative Activity Questionnaire [42]

Changes observed in two clinical and behavioral indicators in a random sample of eligible patients by comparison group exposed to CVD primary prevention actions that emerge from the compared de-implementation strategies, stratified by age and sex. Specifically, a sample of 180 exposed patients will be selected, 15 patients per stratum (2 sex strata, 3 age strata, 2 de-implementation strategies). The use of the aforementioned sampling scheme reflects the intention to maximize the comparability of the populations of exposed users.

As an objective clinical measurement, the change in the REGICOR estimate 12 months after the exposure to the CVD prevention intervention.

As a subjective measurement, the self-reported changes in lifestyle (physical activity, diet, and smoking) 12 months after the intervention, measured through the “prescribe healthy life” (PVS) questionnaire [43], operationalized as (i) proportion of people who have achieved the recommended levels of physical activity (150 min/week of moderate physical activity or 75 min/week of intense physical activity), healthy diet (5 portions/day of fruits and vegetables), and smoking cessation, among those who did not meet these recommended levels on entry into the study; and (ii) weekly minutes dedicated to physical activity of at least moderate intensity, daily number of fruit, and vegetable servings.

#### Adoption

Percentage of FPs out of the total addressed to participate that collaborate in a process to optimize CVD primary prevention practice; characteristics of those participating/not-participating.

Percentage of FPs who change their CVD prevention practice by reducing low-value statin prescribing and increasing health promotion actions in the target population at risk of receiving CVD prevention, 12 months after exposure of physicians to the compared de-implementation strategies.

#### Implementation (main outcome)

The main outcome measures will compare the incidence of both the pharmacological prescribing of statins and the health promotion actions in patients of the target population at risk of receiving CVD primary prevention care, over the 12 months after exposure of collaborating physicians to the compared de-implementation strategies. Specifically, the two following indicators will be compared:

- Incidence of low-value prescriptions of statins in patients with no history of statin prescriptions aged between 40 and 74 years in men and between 45 and 74 years in women, with hypercholesterolemia but without ischemic heart disease/CVD, and with an estimated CVD Risk < 5%, attending during the 12 months after exposure of physicians to the compared implementation strategies
- Incidence of advice regarding healthy lifestyles in patients with no history of statin prescriptions aged between 40 and 74 years in men and between 45 and 74 years in women, with hypercholesterolemia but without ischemic heart disease/CVD, and with an estimated CVD Risk < 5%, attending PC consultations during the 12 months after exposure of physicians to the compared implementation strategies.

#### Maintenance

Incidence of low-value pharmacological prescriptions of statins and of advice regarding healthy lifestyles in eligible patients, 36 months after exposure of physicians to the compared implementation strategies

#### Spreading

With the aim of assessing a possible spill-over effect of the compared de-implementation strategies in the optimization on the CVD prevention practice, de-prescribing rates in patients with a low-value prescription of statins prescribed before the exposure of physicians to the de-implementation strategies

### Fidelity and feasibility measures

A complete recording and subsequent description of the execution process of the compared de-implementation strategies by group will be carried out to assess the level of fidelity with which they have been carried out with respect to what has been planned: number and percentage of professionals included among those addressed to participate; description of actions carried out (training, work sessions, etc.) and final duration compared to what was planned; participation of professional collaborators in each action (exposure); assessment of the content and usefulness of the implementation actions received by the clinicians; resources dedicated to the execution of de-implementation strategies: support materials, external resources (e.g., external facilitator); and organizational support resources (release of professionals, coverage of services, etc.). All changes made to the de-implementation strategy will be thoroughly described: what has been modified, how, why, by whom, and at what level of delivery (e.g., comparison group level).

### Management, quality, and safety in data processing

All data related to patient sociodemographic characteristics (age, sex, socioeconomic status, etc.) and the clinical practice of their healthcare professionals (e.g., pharmacological prescribing, heath promotion actions) will be extracted from the electronic health record system of Osakidetza (OSABIDE). The Primary Care Research Unit of Bizkaia is formally authorized to extract and use data from electronic health records for research purposes by the Healthcare Directorate of Osakidetza. The confidentiality of the people included in the study will be safeguarded at all times, in accordance with the provisions of the Spanish Organic Law on Protection of Personal Data (3/2018 December 5th, LOPD).

### Analysis

Frequencies and proportions along with their corresponding 95% confidence intervals (CIs) will be used to describe the prevalence and the incidence of low-value pharmacological prescribing of statins in primary prevention of CVD of PC professionals (2018 prescriptions being used as the reference level). Factors associated with inappropriate prescribing of statins will be analyzed through stratified statistical analysis and fitting linear logistic models. Given the three levels involved (patients, clinicians, and health organizations), the average variability and possible associated factors will be estimated using hierarchical models and models based on Bayesian estimators.

The primary outcome will be the incidence of new inappropriate prescriptions of statins in patients of the target population (hypercholesterolemic patients without past or current CVD and with an estimated 10-year cardiovascular risk < 5%) 12 months after exposure of physicians to the compared de-implementation strategies. Therefore, to evaluate the impact of the alternative de-implementation strategies, the relative risk of receiving an inappropriate prescription of statins in patients in the target population of the experimental intervention over that of patients from the reference/control group will be estimated by intention-to-treat. Change in new inappropriate prescription incidence rates from baseline to those observed 12 months after physicians’ exposure to the de-implementation strategies and the relative risk reduction ratio will be estimated with the corresponding 95% confidence intervals. In order to adjust for potential confounding factors, stratified statistical analyses and logistic models will be used. These models will be extended to generalized mixed-effects models to take into account the hierarchical structure of data (patients nested in physicians and physicians in PC teams or specialized care services), with fixed effects (comparison group, effect of time on outcome indicators, and time-group interaction) and random effects on the intercept and the time slope (for each patient, physician, center, etc.). These models will be adjusted for potential confounding variables, following a “forward” strategy guided by the stratified analyses. A similar analytic strategy will be followed for secondary outcomes. The analyses will be carried out with SAS, Stata, and R.

Based on (i) a baseline incidence of 2.7% of new inappropriate statin prescriptions estimated among the patients of the target population seen in 2018 by physicians with an inadequate prescription incidence > 0% with a minimum cluster size n ≥ 20 patients, (ii) an intra-class correlation coefficient of 0.01, (iii) an average cluster size of 56 patients with a coefficient of variation of 0.43, (iv) α = 0.05 and statistical power of 80%, and (v) a hypothetical reduction in annual inappropriate prescription rates in the intervention group of 1.7% in absolute terms or 63% in relative terms (from 2.7 to 1%), the interventional phase will require two study groups with at least 22 physicians each, i.e., at least 44 PC physicians.

## Discussion

The DE-imFAR study aims to provide valid scientific knowledge regarding a structured and theory-informed process for designing and evaluating effective and feasible de-implementation strategies to favor the abandonment of low-value pharmacological prescribing in clinical practice. In brief, the study proposes a detailed systematic process for identifying determinants of low-value practices, identifying behavioral objectives as areas for improvement, designing, and operationalizing de-implementation strategies informed by several theoretical frameworks to target the identified multilevel determinates of low-value practices, and finally, evaluating the feasibility and potential effectiveness of the de-implementation strategies to reduce the low-value practice. Specifically, the present study targets the unnecessary pharmacological prescribing of statins in the primary prevention of CVD in low-risk patients.

There is a growing body of evidence suggesting that the elimination (that is, the de-implementation) of ineffective and potentially harmful clinical practices (i.e., low-value) is essential for the provision of high-quality care and the sustainability of our health system. Nonetheless, such initiatives are being limited to producing lists of candidate practices to be eliminated and/or of what-not-to-do recommendations. The evidence base related to successful de-implementation strategies to eliminate low-value practices is in its infancy.

This study aims to provide valid scientific knowledge in several specific areas related to the de-implementation of low-value practices. First, this is among the first studies applying the most prominent implementation theories and frameworks specific to the field of de-implementation. In doing so, it aims to compare the effectiveness and feasibility of passive dissemination-based interventions with active de-implementation strategies that emerge from a structured and theory-informed process for the design of de-implementation strategies. Second, due to the clinical scenario addressed, the reduction of low-value prescribing of statins in CVD primary prevention where the promotion of healthy lifestyles is the recommended practice, the present project will attempt to shed light on the added effect of replacement with an alternative for the de-implementation of a given low-value care, over reversal without a substitute or alternative. And third and last, the eventual expected impact of this project is to tackle a type of low-value intervention that has a significant impact on health costs for our health system and for patients themselves, thereby contributing to the financial sustainability of the health system. All of this is to be achieved through robust research into new practices and innovations for the optimization of clinicians’ practice and the provision of health services, guided by the emerging science of implementation and de-implementation. If the strategies explored are successful, health planners and managers will have the evidence needed to support the introduction of innovations emerging from research in these innovative fields.

## Data Availability

Since data supporting the present study will mostly concern routine data retrieved from the electronic health records of the Basque Health Service-Osakidetza, it will be only shared on justified request to the study guarantors.

## Abbreviations

BCW: Behavior Change Wheel
CFIR: Consolidated Framework for Implementation Research
CVD: Cardiovascular disease
FP: Family physician
IHO: Integrated Healthcare Organizations
PC: Primary care
TDF: Theoretical Domains Framework
